# Parents' Emotional Journey Throughout Their Participation in a Well‐Being Support Group Intervention

**DOI:** 10.1111/jar.70107

**Published:** 2025-08-07

**Authors:** Mélina Boulé, Mélina Rivard, Catherine Mello

**Affiliations:** ^1^ Department of Psychology University of Quebec in Montreal Montreal Quebec Canada; ^2^ Department of Psychology The Pennsylvania State University University Park Pennsylvania USA

**Keywords:** emotional well‐being, intellectual and developmental disabilities, interventions, journaling, parents

## Abstract

**Background:**

Family carers often lack support for their own well‐being as they navigate diagnostic and early intervention services for intellectual and developmental disabilities.

**Methods:**

This study explored the emotional journey of carers during Early Positive Approaches to Support, an 8‐week group program. Participants journaled their emotional experiences at the beginning and end of each session.

**Results:**

There was a significant increase in comfortable emotions and a decrease in uncomfortable emotions by the program's end.

**Conclusions:**

These findings suggest that the program improved carers' ability to self‐regulate their emotions and manage challenges. This study also highlights the value of self‐observation to understand carers' emotional landscapes in interventions targeting their well‐being.

**Summary**

- This study focused on the emotional journey of family carers navigating diagnostic and early intervention services for their child with intellectual and developmental disabilities.
- Over the course of an 8‐week support group program carers experienced an increase in comfortable emotions and a decrease in uncomfortable emotions among carers.
- Research typically focuses on the challenges faced by carers, yet these often coexist with positive experiences. In this study, carers consistently reported more frequent and intense comfortable emotions than uncomfortable emotions.
- This study underscores the importance of self‐observation through journaling as a tool to explore and understand carers' emotional experiences in emotions aimed at enhancing well‐being.

## 1 Introduction

The experience of raising a child with intellectual and developmental disabilities^1^ often differs significantly from raising typically developing children (Fairthorne et al. 2018; Savari et al. 2023). Family carers face a unique array of challenges, joys, roles, and responsibilities including considerable daily accommodations as they support their child's development (Higgins et al. 2023; Nurullah 2013; Papadopoulos 2021). From the moment a family member, paediatrician, or childcare professional suspects an intellectual and/or developmental delay, parents navigate through multiple steps as they request and await interventions (Grenier‐Martin and Rivard 2020; Rivard et al. 2021, 2023). Due to a lack of emotional, informational, and practical support, they frequently feel isolated and powerless to meet their child's additional needs, which can lead to the development of less functional coping strategies, such as experiential avoidance or expression emotional suppression (Avdiu and Hyseni Duraku 2024; Gur and Reich 2023). This context may contribute to the onset of mental health difficulties (Masefield et al. 2020; Savari et al. 2023).

Some interventions designed to support parents' psychological well‐being have shown promising impacts on standardised outcome measures of parenting stress, depression, and anxiety (Bourke‐Taylor et al. 2021; Kulasinghe et al. 2023). These interventions typically integrate strategies based on positive approaches such as Acceptance and Commitment Therapy or mindfulness strategies (e.g., Cachia et al. 2016; Chan and Neece 2018; Conrad et al. 2021; Kabat‐Zinn 1990; Hayes et al. 1999). However, these interventions are rarely accessible to family carers through public health and social care systems (Derguy et al. 2017; Rivard et al. 2023). Some interventions may offer information or coaching. However, these approaches primarily aim to enhance parental knowledge and adjust parenting practices, rather than directly targeting parents' emotional and psychological well‐being (Frantz et al. 2018). There are significant costs to seeking help from private services due to a lack of public options (Shepherd et al. 2020). To the extent that parents already face challenges in accessing high‐quality interventions for their child, they may prioritise their child and postpone or avoid seeking out formal resources for their own well‐being (Fairthorne et al. 2018; Papadopoulos 2021).

With the goal of increasing family carers' access to interventions that promote their emotional and psychological well‐being, a community‐based participatory research initiative was undertaken to evaluate and implement the Early Positive Approaches to Support (E‐PAtS; Gore et al. 2022) program within public health and social service agencies in Quebec, Canada. This group program provides informational, psychoeducational, and emotional support as well as a safe space for family carers to share their personal experiences of raising a child with intellectual and developmental disabilities and navigating services. As part of this initiative, the present study specifically focused on the impacts of E‐PAtS on parents' emotional experience. This research provides unique insights into the impact of the program and on the relevance of a self‐observation method to assess emotional outcomes in program evaluation and research. Emotional experiences are seldom assessed as intervention outcomes, even when these are the target of interventions. Furthermore, few studies use longitudinal observational designs that could shed light on emotional evolution over the duration of the intervention.

### 1.1 Assessment of Emotional Experience in Program Evaluation

Research on mental health in parents has primarily focused on the negative consequences of raising a child with intellectual and developmental disabilities for parents, as measured by a global index, such as high levels of anxiety, depressive symptoms, and reduced quality of life (Higgins et al. 2023). However, despite the challenges these families face, positive experiences and beneficial effects can emerge from these unique circumstances, such as increased life satisfaction, gratitude, and hope (Halstead et al. 2018; Meleady et al. 2020; Ranjan et al. 2023). In studies with qualitative design, parents report experiencing feelings of guilt, disappointment, anger, grief, fear, and powerlessness in relation to the assessment process or the manifestations of their child's diagnosis and its implications for the child's future (Nurullah 2013; Ooi et al. 2016; Ranjan et al. 2023). Yet, these challenging emotions often coexist with positive feelings such as joy, hope, gratitude, pride, and satisfaction (Boulé and Rivard 2024; Higgins et al. 2023; Ranjan et al. 2023). Parents' emotional regulation is suggested to play an important role in parenting and child development (Aydin 2023; Zimmer‐Gembeck et al. 2022). This suggests that program evaluation should seek to incorporate measures of emotional experience (e.g., an emotional journal) alongside typically assessed outcomes.

As mentioned, parents may engage in emotional avoidance or suppression, which poses challenges in selecting measures that accurately capture their emotional experiences (Avdiu and Hyseni Duraku 2024; Gur and Reich 2023). One strategy to support emotional processing is the use of emotion journals, which can help parents identify, express, and reflect on their feelings. Prior research has documented the clinical benefits of emotion journaling across diverse populations, highlighting its positive effects on mental health outcomes (e.g., Dimitroff et al. 2017; Ullrich and Lutgendorf 2002). A recent meta‐analysis further demonstrated that emotional journaling represents a low‐cost, low‐risk tool with potentially significant clinical impact across various settings (Sohal et al. 2022). In the context of intellectual and developmental disabilities, no studies to our knowledge have systematically documented parents' emotional experiences through self‐observation data or examined how interventions that target psychological well‐being impact this emotional landscape (Frantz et al. 2018; Shepherd et al. 2020).

Self‐report measures may be advantageous in situations that require a comprehensive evaluation of a program's full impact on emotional and psychological well‐being (Kaplan et al. 2012). Emotions, as perceptual and self‐referential phenomena, are most accurately captured by the person experiencing them, at the moment they are experiencing them. Thus, such self‐reported data are considered more valid (Robinson and Clore 2002). Unlike external observational methods, self‐report measures do not rely on emotional states being visibly expressed or on an observer's interpretation of such expressions (Chan 2010). However, this approach requires that individuals can recognise and label their emotions. An emotional journal can help by proposing a vocabulary and inviting the individual to reflect on their internal experience (Kaplan et al. 2012). This approach supports repeated measurement throughout a program, which would facilitate tracking improvements in this ability.

Mauss and Robinson (2009) suggest that emotional measures tend to be structured along dimensions (e.g., valence, arousal) rather than discrete emotional states (e.g., sadness, fear, anger). This provides a framework to analyse how participants experience and regulate their emotional state over time on a continuum ranging from comfortable to uncomfortable and of varying intensity levels (Mauss and Robinson 2009). This type of assessment could provide a more nuanced view of participants' emotional growth and self‐perception, which could be a useful complement to traditional program evaluation approaches centred on mental health indicators and qualitative evaluations (Mauss and Robinson 2009; Kaplan et al. 2012).

### 1.2 Integration of the E‐PAtS Program in Community‐Based Services

In Quebec (Canada), as well as elsewhere in the world, most supports for intellectual and developmental disabilities within public health and social services agencies focus on direct intervention for the child, either as individual interventions with a therapist or through parental coaching where family carers apply intervention strategies at home (Grenier‐Martin and Rivard 2020; Rivard et al. 2021, 2023; Shepherd et al. 2020). Although parents express a need for support (Ranjan et al. 2023; Shepherd et al. 2020), public services do not typically offer interventions to promote their well‐being and help them to navigate the steps and challenges associated with their child's diagnosis or needs. To bridge this gap, E‐PAtS was considered for integration within Quebec's public care and services trajectory for intellectual and developmental disabilities. The addition of this program would help promote family carers' well‐being as early as possible in the child's diagnostic and services journey (see Rivard et al. in revision).

E‐PAtS is a transdiagnostic group intervention developed and rigorously evaluated by, for, and with parents, practitioners, and researchers in the United Kingdom (see Coulman et al. 2022; Gore et al. 2022). The program is structured to address the needs of families with children aged up to 5 years old who have a wide range of intellectual and developmental delays or disabilities. The program is co‐facilitated by a dyad comprising a professional and a parent who have both completed the same E‐PAtS training. Each session addresses a theme identified as relevant by families and informed by empirical data. Session 1 focuses on navigating available services and supports, Session 2 addresses the importance of self‐care for parents, Session 3 explores sleep, Session 4 focuses on communication, Session 5 covers adaptive behaviours, Sessions 6 and 7 address challenging behaviours, and Session 8 serves as a closing and reflection session. E‐PAtS consists of eight weekly sessions delivered online or in person, each 2–2.5 h long, that combine activities, psychoeducational content, and discussions.

While mental health research has historically focused on psychological dysfunction, there is increasing recognition of the importance of also promoting well‐being (Bohlmeijer and Westerhof 2021; Ryff and Singer 1996). Interventions that validate emotional experiences, both positive and difficult, can enhance emotional awareness and regulation, and foster greater acceptance of internal experiences (Hayes et al. 1999). By offering a non‐judgmental space for parents to share diverse experiences related to caring for a child with an intellectual or developmental disability, and by drawing on positive psychological approaches, the E‐PAtS program supports parents in navigating their emotional experiences and strengthening resilience within the family context (Gore et al. 2022). Therefore, E‐PAtS aims to give parents access to evidence‐based services, empower them, help them manage their child's needs, and build their emotional resilience (Gore et al. 2022).

In the United Kingdom, the E‐PAtS program recently underwent a feasibility randomised controlled trial (Coulman et al. 2022) in preparation for a definitive randomised controlled trial, which is presently underway. Parent mental health and family functioning outcome data collected as part of this feasibility evaluation was suggestive of favourable changes in the intervention group (Coulman et al. 2022). In parallel, a qualitative study describing family experiences (Gore et al. 2022) indicated that parents perceived E‐PAtS as a supportive and empowering intervention that fosters a sense of connection, increased understanding of their child's needs, and enhanced coping strategies (Gore et al. 2022). The adaptation and implementation of E‐PAtS in a new linguistic and cultural context, outside of the United Kingdom, are currently being evaluated within public services in Quebec.

This broad study evaluating E‐PAtS in a different context to support parental well‐being offers a valuable opportunity to explore the relevance of using a self‐reported emotion measure, both from a clinical and methodological perspective, within such an intervention. To date, no study on E‐PAtS or similar programs focused on emotional well‐being has systematically documented the evolution of parents' emotional experience, namely the type and intensity of their emotions, as they complete a program. The present study, as part of the broader evaluation of E‐PAtS in Quebec (Canada), aimed to address the challenge of capturing these complex internal experiences, which are difficult to measure with traditional mental health questionnaires, by incorporating self‐observation measures.

### 1.3 Objectives

The present study sought to examine parents' emotional experiences throughout their participation in E‐PAtS. It employed an emotional journaling tool to explore the following questions:

1. Which emotions do parents report experiencing before and after a session, and at what intensity?
2. How do the number and intensity of reported emotions change during sessions and over the duration of the program, depending on their valence?
3. Are there specific sessions that seem to have a greater influence on the valence, quantity, or intensity of reported emotions?

## 2 Method

The larger study on E‐PAtS in which the present research was conducted received approval from the Research Ethics Boards of the University of Quebec in Montreal as well as the Research Ethics Committee of Integrated University Health and Social Services Centre of Mauricie‐et‐du‐Centre‐du‐Québec.

### 2.1 Participants

Participants were recruited through the waiting lists for early intervention services at four public health and social service agencies in different regions of Quebec (two regional agencies, one specialised paediatric hospital, and one community center). To participate in the program and in this study, parents had to speak English or French. Their child had to be aged 7 or under and have an intellectual and developmental disability (diagnosed or suspected), which corresponds to the eligibility criteria for families to receive specialised early childhood services in the public network of Quebec. Two carers were invited to participate in each family. A total of 104 participants, across 19 cohorts, received the E‐PAtS program. All written consents were obtained from participants for their participation in the program as well as in the research project.

Table 1. lists detailed demographic information for participating families. Most (*n* = 101; 93%) participants were mothers and eight were fathers (7%). More than half of the parents were born in Canada (59%), while the other participants were primarily from Africa (24%) or other regions of the Americas outside Canada (14%). The majority of participants lived in a nuclear family (71%). Nearly equal proportions of parents reported an annual household income below CAN$30,000 (23%), between $30,000 and $50,000 (24%), or between $50,000and $70,000 (20%). Twelve percent of participants had an annual income exceeding $120,000. A larger portion of parents had a 2‐ or 3‐year postsecondary degree (44%), while a similar number either had a high school degree (27%) or had pursued university studies (27%). Most parents were employed full‐time (41%), while 30% were unemployed. Their children were aged between 2 years 6 months and 7 years 9 months (*M* = 4.3; SD = 1.3). The majority of participants' children (65%) were either in the process of being evaluated or awaiting evaluation for a suspected intellectual or developmental disability, whereas 35% had a confirmed diagnosis of either autism (*n* = 9), autism and intellectual disability (*n* = 5) attention deficit hyperactivity disorder (*n* = 4), intellectual disability (*n* = 3), or speech or language delay (*n* = 3).

**TABLE 1:** Participants' sociodemographic data (*N* = 104).

	*n*	Missing (%)	%
Type of family	79	20.2%	
Nuclear			70.9
Separated or divorced			10.1
Stepfamily			6.3
Single parent			12.7
Annual household income (CAD)	84	15.2%	
$0–29,999			22.6
$30,000–49,999			23.8
$50,000–69,999			20.3
$70,000–89,999			8.3
$90,000–119,999			13.1
$120,000+			11.9
Parents' level of education	84	15.2%	
High school or lower			27.4
DCS/DVS^a^			44.0
University			28.6
Parents' employment status	84	15.2%	
Full‐time, salaried employee			40.5
Part‐time, salaried employee			14.3
Contract worker			7.1
Not working outside the home			29.8
Other (on leave, student)			8.3
Parents' place of birth	82	17.2%	
Canada			58.5
Americas, except Canada			13.5
Africa			24.4
Asia and Middle East			1.2
Europe			2.4
Oceania			

^a^ In Québec, a diploma of college studies (DCS) is a postsecondary degree in preparation for university‐level education or a trade; the diploma of vocational studies (DVS) is a secondary degree in preparation for a specialised occupation.

### 2.2 Measures

#### 2.2.1 Sociodemographic Questionnaire

A sociodemographic questionnaire was used to gather information on household income and parents' employment status, educational background, country of origin, and primary language spoken at home. This questionnaire also collected information on the child's diagnosis, age, and services received, and on family structure, such as the number of siblings.

#### 2.2.2 Emotional Journal

For this study, an online emotional journal was developed as the primary data collection tool to capture participants' emotional experiences over the course of the 8 weeks of the E‐PAtS program. Participants were asked to complete the journal at the beginning and end of each weekly session, that is, totalling 16 entries. Participants received a link to the journaling tool, hosted on the Qualtrics survey platform, through a chat box (for groups that met virtually) or by email (for groups that met in person). A research assistant ensured that participants could access the tool, which required less than 5 min to use.

The development of the emotional journal was informed by a review of the literature on emotional measurement tools and terminology conducted by the research team. The journal included an equal number of comfortable (e.g., happy, grateful, motivated) and uncomfortable (e.g., tired, angry, sad) emotions to balance valence representation (Mauss and Robinson 2009). Two psychologists and two researchers initially selected the emotions. The tool was then reviewed and validated by the entire research team, which included clinicians and parents as well as academic researchers. The final journal featured a list of 20 emotions and two ‘Other’ options for participants to type in any emotions not listed. Participants were instructed to select all emotions they were experiencing and rate the intensity of each on a 3‐point Likert scale (*a little*, *somewhat*, and *a lot*).

### 2.3 Procedure

The research team partnered with public agencies that would provide a venue for recruitment and delivery of the program. E‐PAtS was offered to families awaiting evaluation (i.e., their child was suspected of having a developmental disability) or intervention services (i.e., their child had received a diagnosis). Each centre provided at least one trained professional facilitator to lead the program. Professional facilitators were psychologists, social workers, psychoeducators, and speech and language therapists. They were paired with parent facilitators identified by the centre or members of the research team. Facilitators were selected for E‐PAtS training based on their personal and interpersonal qualities, particularly their capacity for empathy, openness, and collaboration, as well as their ability to embody the core principles of the program. A guidance document outlining these key characteristics was provided to each centre to support the identification of professionals and parents who matched the desired facilitator profile. Both types of facilitators received the same five‐day training, which covered the full content of the program, included opportunities for practice and constructive feedback, emphasised the core principles of the program, and highlighted the importance of facilitator stance and interpersonal presence. The training also aimed to prepare facilitators for effective co‐facilitation. Parents completed the sociodemographic questionnaire at program intake, typically in the week preceding the first session. Over the course of 8 weeks, the professional and parent facilitator pair held weekly sessions according to their availability. Most groups (16) met virtually (using videoconferencing software) and three groups met in person. For more detailed information regarding the deployment and delivery of E‐PAtS, see Rivard and colleagues (in revision).

### 2.4 Statistical Analyses

Descriptive analyses (i.e., frequencies, means, and standard deviations) were computed for each emotion in each of the 16 journal entries (i.e., before and after the 8 sessions). The 20 emotions listed in the journal were categorised as ‘comfortable’ and ‘uncomfortable’ to simplify the analysis of emotional valence. Although the journal provided open‐ended response options, upon examination by the research team, these were not included in analyses as most of these entries reflected facts, events, or thoughts (e.g., ‘headache’, ‘normal’, ‘cold’) rather than emotions that could be categorised in terms of valence. First, to evaluate changes between the beginning (pre‐Session 1) and end of the program (post‐Session 8), two mixed‐effects models were performed on the number and intensity of emotions, respectively, as a function of Program Completion (i.e., beginning vs. end) and Valence (comfortable or uncomfortable; fixed effects) while accounting for variability between participants (random effect). Second, to capture session‐specific changes, a third fixed effect (Session: 1 through 8) was added to these two models. For the present study, this analytical approach was preferred to repeated‐measures analyses of variance (ANOVAs) because it enables all available data to be taken into account despite missing data (e.g., participants who missed a session); however, for ease of interpretation, we report these results in a manner analogous to ANOVA.

## 3 Results

### 3.1 Type and Intensity of Emotions Reported Before and After Sessions

Table 2 presents the mean intensity of each emotion reported in pre‐ and post‐session journal entries for all eight sessions taken together. A total of 2053 distinct emotions were reported before sessions and 2068 after sessions, totaling 4121 emotions. Before sessions, participants most often expressed interest (*n* = 302; 19% of all comfortable emotions reported). Fatigue represented more than half of all uncomfortable emotions both before (55%) and after (66%) sessions, but these entries were less frequent at the end of sessions (before, *n* = 266; after, *n* = 209). The frequency of optimism was similar before (*n* = 166) and after sessions (*n* = 167). Feelings of satisfaction were most frequently expressed after sessions (*n* = 229) compared to before (*n* = 104). Before sessions, participants also frequently reported feeling happy (*n* = 199), motivated (*n* = 183) and confident (*n* = 172) and least frequently reported feeling concerned (*n* = 25), powerless (*n* = 16), or discouraged (*n* = 11). After sessions, participants primarily reported confidence (*n* = 225) and gratitude (*n* = 200), whereas powerlessness (*n* = 2) and anger (*n* = 1) were rarely mentioned.

**TABLE 2:** Frequency and intensity of emotions reported across all pre‐ and post‐session journals.

	Pre				Post			
	Frequency		Intensity		Frequency		Intensity	
	*n*	%	*M*	SD	*n*	%	*M*	SD
Comfortable emotions	1571	76.5	2.44	0.54	1749	84.6	2.56	0.52
Happiness	199	12.7	2.36	0.52	213	12.2	2.49	0.53
Curiosity	143	9.1	2.51	0.55	81	4.6	2.57	0.50
Calm	53	3.4	2.13	0.59	123	7.0	2.32	0.62
Satisfaction	104	6.6	2.38	0.53	229	13.1	2.52	0.54
Optimism	166	10.6	2.40	0.60	167	9.5	2.54	0.55
Confidence	172	10.9	2.4	0.57	225	12.9	2.54	0.53
Interest	302	19.2	2.63	0.50	214	12.2	2.69	0.47
Enthusiasm	127	8.1	2.43	0.57	117	6.7	2.56	0.53
Gratitude	122	7.8	2.66	0.47	200	11.4	2.76	0.43
Motivation	183	11.6	2.51	0.52	180	10.3	2.58	0.53
Uncomfortable emotions	482	23.5	1.92	0.79	319	15.4	2.01	0.74
Fatigue	266	55.2	2.03	0.77	209	65.5	2.17	0.72
Anger	10	2.1	1.70	0.67	1	0.3	n/a	n/a
Disappointment	8	1.7	1.88	0.83	13	4.1	1.15	0.38
Sadness	19	3.9	1.95	0.78	19	6.0	1.84	0.90
Worry	54	11.2	2.02	0.84	25	7.8	2.24	0.78
Shame	3	0.6	2.00	1.00	2	0.6	2.50	0.71
Loneliness	21	4.4	2.00	0.71	10	3.1	2.20	0.79
Powerlessness	37	7.7	1.95	0.78	16	5.0	2.25	0.77
Discouragement	46	9.5	1.87	0.81	11	3.4	1.91	0.83
Shyness	18	3.7	1.78	0.73	13	4.1	1.85	0.80

Gratitude had the highest mean intensity both before (*M* = 2.66, SD = 0.47) and after (*M* = 2.76, SD = 0.43) sessions. Anger had the lowest intensity before sessions (*M* = 1.70, SD = 0.67) and was only reported once, with an intensity of 2 (i.e., *somewhat*), after a session.

### 3.2 Emotions Reported Before and After the Intervention

There was a significant main effect of Valence, *F*(1, 205.41) = 147.34, *p* < 0.001, on the number of emotions journaled immediately before (i.e., before Session 1; *M* = 1.88) and after the program (i.e., after Session 8; *M* = 1.74), although the main effect of Program Completion did not meet statistical significance, *F*(1, 254.34) = 3.79, *p* = 0.053. These were qualified by a significant Program Completion by Valence Interaction, *F*(1, 205.41) = 24.93, *p* < 0.001. By the end of the program, compared to the beginning, the number of comfortable emotions increased significantly (*M* = 2.53–3.07, SD = 2.11 and 2.24, *p* = 0.038) whereas uncomfortable emotions had decreased significantly (*M* = 1.22–0.41, SD = 1.21 and 0.74, *p* < 0.001). At both time points, comfortable emotions outnumbered uncomfortable emotions.

This interaction was not observed for the intensity of emotions, *F*(1, 153.63) = 0.463, *p* = 0.497. Participants reported significantly more intense emotions, irrespective of valence, at the end of the program (*M* = 2.53, SD = 0.58) relative to the beginning (*M* = 2.18, SD = 0.66), *F*(1, 156.56) = 7.18, *p* = 0.008. Comfortable emotions (*M* = 2.54, SD = 0.48) were rated as significantly more intense than uncomfortable ones (*M* = 1.94, SD = 0.71), *F*(1, 154.73) = 53.74, *p* < 0.001.

### 3.3 Emotions Reported Across Sessions Throughout the Program

**FIGURE 1:**
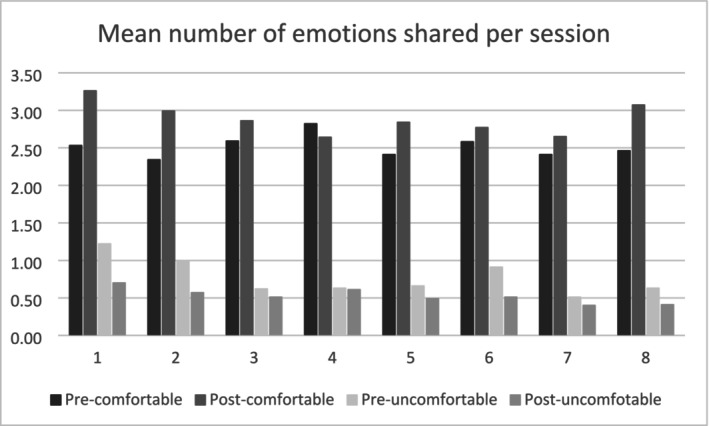
Mean of number of emotions journaled per session. Pre‐ and post‐ refer to emotional journaling that took place immediate before and after each session.

From the 2053 emotions reported in pre‐session journals, 76.5% were categorised as comfortable (*n* = 1571) and 23.5% were uncomfortable (*n* = 482). This difference was amplified in post‐session entries to 84.6% (*n* = 1749) comfortable and 15.4% (*n* = 319) uncomfortable (see Table 2 and Figure 1) emotions.

Table 3 presents detailed results for the number of emotions across each of the 8 sessions. The analysis of the number of comfortable and uncomfortable emotions (Valence) reported before and after (Time) for Sessions 1–8 (Session) yielded significant main effects of Valence, *F*(1, 2289.56) = 1611.45, *p* < 0.001, and Session, *F*(1, 2311.56) = 2.42, *p* = 0.018, and a significant Valence * Time interaction, *F*(1, 2289.56) = 41.66, *p* < 0.001. Because the three‐way interaction was also significant, *F*(1, 2289.56) = 2.09, *p* = 0.005, separate analyses were run for each session. The main effect of Valence was observed across all 8 sessions, indicating that comfortable emotions (*M* = 2.70, SD = 2.21) consistently outnumbered uncomfortable (*M* = 0.66, SD = 0.93) emotions. In general, post‐session measures displayed an increase in the number of positive emotions (overall, *M* = 2.52–2.89, SD = 2.14 and 2.26) but a decrease in uncomfortable emotions (overall, *M* = 0.78–0.53, SD = 1.03 and 0.79), relative to the corresponding pre‐session measure.

**TABLE 3:** Number of emotions reported across time and sessions based on valence.

Session	Comfortable				Uncomfortable			
	Pre	Post	Difference	*p*	Pre	Post	Difference	*p*
1	2.53	3.25	0.210	**0.011**	1.22	0.69	−0.257	**0.002**
2	2.34	2.99	0.229	**0.004**	0.99	0.57	−0.216	**0.006**
3	2.59	2.86	0.058	0.453	0.62	0.51	−0.039	0.611
4	2.82	2.64	−0.055	0.541	0.63	0.61	0.008	0.924
5	2.41	2.84	0.169	**0.025**	0.66	0.49	−0.079	0.292
6	2.58	2.75	0.095	0.306	0.91	0.52	−0.181	**0.043**
7	2.41	2.65	0.067	0.420	0.51	0.40	−0.081	0.328
8	2.46	3.07	0.187	**0.026**	0.63	0.41	−0.121	0.148

*Note:* Bold values indicate statistically significant results.

The analysis of the intensity of uncomfortable emotions yielded significant main effects of Valence, *F*(1, 1529.55) = 296.47, *p* < 0.001, and Time, *F*(1, 1491.37) = 18.18, *p* < 0.001, which were qualified by a significant Valence * Time interaction, *F*(1, 1495.25) = 3.91, *p* = 0.048.

Participants reported less intense emotions before, compared to after, sessions, but this effect was less pronounced for comfortable (*M* = 2.44 vs. 2.49, SD = 0.48 and 0.47, *p* = 0.045) relative to uncomfortable emotions (*M* = 1.94 vs. 2.11, SD = 0.70 and 0.69, *p* < 0.001). There was no main effect or interaction involving Session, all *p* ≥ 0.184.

## 4 Discussion

Family carers of young children with intellectual and developmental disabilities often experience a complex range of emotions that can be difficult to express in their social circles or to healthcare and educational personnel (Nurullah 2013; Staunton et al. 2023). In addition to handling the daily demands of their lives (e.g., parental responsibilities, work, medical appointments, coordinating various services), parents attempt to make sense of, and cope with, their internal emotional experiences. With limited access to formal services or informal social support, it is unsurprising that their own well‐being often takes a backseat as they focus their energy on caring for their child and advocating for services (Andreyko 2016; Camard et al. 2023; Ranjan et al. 2023). Parents' emotional and psychological well‐being can significantly impact their capacity to implement interventions and how they interact with their child. This, in turn, may influence the child's prognosis and thus contribute to parental feelings of guilt, powerlessness, and anxiety (Fang et al. 2024; Suvarna et al. 2024).

The E‐PAtS program is intended to create a safe space for family carers to share and process their feelings. They can openly discuss how they feel about the experience of raising a child with intellectual and developmental disabilities among their peers without fear of judgement (Gore et al. 2022). In the present study, participants took the time to reflect on their emotions by filling out an emotional journal immediately before and after each of their eight weekly sessions. This addition to the E‐PAtS experience could further help parents recognise, name, and share their emotional experiences within the supportive group setting. The results of this study also illustrate the strength of the journal not only as part of the intervention but as a powerful measure of the internal processes that take place throughout the intervention.

### 4.1 Emotional Changes Throughout E‐PAtS

Comfortable emotions represented approximately three‐quarters of all emotions expressed by parents throughout the program and consistently outnumbered uncomfortable emotions in every session. This aligns with research indicating that positive or comfortable emotions tend to occur more frequently and with greater intensity than negative emotions in the general population (Zelenski and Larsen 2000). While much of the research on carers of children with intellectual and developmental disabilities has historically focused on the challenges and negative emotional impacts they face, a growing body of literature highlights the co‐occurrence of positive emotions (Boulé and Rivard 2024; Ranjan et al. 2023). Despite discussing personal challenges related to their child's condition in the context of E‐PAtS, participants in the present study consistently expressed more comfortable emotions, including happiness, motivation, and optimism. Gratitude emerged as the emotion journaled with the highest intensity.

The E‐PAtS program appears to have differentially impacted emotional experiences according to their valence: by the program's end, comfortable emotions had increased, while uncomfortable emotions decreased. While this outcome is promising as evidence of improved emotional well‐being among parents, a stable number of uncomfortable emotions would not necessarily have been a cause for concern. Indeed, a core component of E‐PAtS is the recognition that all personal and subjective experiences, even challenging ones, are valid and can support adjustment to stressful situations (Coulman et al. 2022; Gore et al. 2022). In this context, the findings regarding the number of emotions journaled after the program suggest that parents were not only able to experience a reduction in less comfortable emotions but also to acknowledge and validate their presence rather than disregarding them. Group discussions helped them normalise these emotions and move forward to also recognise positive aspects of their experiences, such as moments of joy. For instance, acknowledging anger over waiting lists for services and reframing such feelings as motivation for advocacy, rather than suppressing them, may empower parents (Camard et al. 2023). This shift in perspective could promote positive emotions such as confidence and hope even though the underlying anger remains. Programs like E‐PAtS create conditions where parents can safely explore the coexistence of comfortable and uncomfortable emotions.

Emotions reported at the end of a session, regardless of their valence, tended to be of greater intensity than those reported before the session. Additionally, comfortable emotions tended to be rated more as intense than uncomfortable ones. Discussing experiences, whether positive or negative, can reactivate associated emotions and thus temporarily amplify their intensity (Duken et al. 2021). Additionally, the peer validation process within the group sessions may also have influenced the intensity of emotions reported by participants. However, future studies should consider implementing a version of the instrument with a finer‐grained response scale. Indeed, the 3‐point Likert scale used may have limited the precision or sensitivity of results and thus underestimated subtle differences in parents' emotional experiences (Matell and Jacoby 1972).

Session‐level analyses indicated that the most salient improvements (i.e., increase in comfortable emotions, decrease in uncomfortable emotions) occurred during Sessions 1 and 2. Session 1 focuses on introducing families to available support services and offers a space for participants to share their personal experiences. Session 2 emphasises the importance of parents' prioritising their own well‐being in order to effectively support their family according to their values (Gore et al. 2022). The significant emotional shifts observed in these sessions likely reflect the key themes addressed—namely, improving access to support and promoting self‐care for parents. As for the other differences observed in sessions, sessions 5 (*fostering life skills through active development*) and 8 (*Bringing it all together*) might emphasise a more positive outlook, aligning with the principles of positive psychology. These sessions focus on the child's strengths and future‐oriented goals, with less discussion of challenging moments. In contrast, sessions 6 and 7 (*Responding to challenges Part 1 and 2*) require parents to confront more difficult topics, specifically addressing challenging behaviours. Overall, this progression reflects a deliberate balance between empowering parents with optimism and equipping them to navigate complex challenges, ensuring a comprehensive and supportive program.

### 4.2 Emotional Regulation and Psychological Well‐Being in Parenting

Adaptive emotional regulation refers to being aware of one's emotions, accurately identifying them, and managing one's emotional responses based on goals or situational demands (Gratz and Roemer 2004). Parents' ability to regulate their emotions plays an important role in the responsiveness of their caregiving behaviours but also their children's emotional regulation and overall social–emotional growth (Aydin 2023; Zimmer‐Gembeck et al. 2022). The increase in the number of comfortable emotions observed by the end of the program suggests that parents developed their ability to identify more positive internal experiences and label their emotions over the course of their participation in E‐PAtS, despite still facing challenges related to their child. Because the first step towards effective emotional regulation involves acknowledging and naming them (Kashdan et al. 2015), we hypothesise that the weekly practice of reflecting on their internal experiences helped parents to enhance this skill.

Given the crucial role of emotional regulation in shaping both the parent's and the child's psychological well‐being, interventions aimed at enhancing parents' ability to recognise their internal experiences could be valuable prevention and support options for families. Ultimately, these approaches seek to empower parents to manage their own emotional responses in order to better support their children's needs and to foster overall family functioning and child development (Zimmer‐Gembeck et al. 2022). During times of stress, pleasant and distressing emotions can overlap. The interaction between well‐being and distress fluctuates based on both external stressors and internal dispositions (McNulty and Fincham 2012). In parents of children with intellectual and developmental disabilities, caregiving stress can disrupt the balance between comfortable and uncomfortable emotions. Effective emotional regulation and self‐care (internal dispositions) are therefore critical.

The emotional experiences of parents of children with intellectual and developmental disabilities are more closely associated with parenting stress than with their child's behavioural challenges (see Kurtz‐Nelson and McIntyre 2017). E‐PAtS emphasises emotional well‐being by fostering self‐acceptance and resilience. In a previous study, parents reported greater awareness of the need for self‐care and adopted small but impactful strategies to improve their well‐being. They also reported enhancements in their mood and self‐perception (Gore et al. 2022). By prompting parents to reflect on their emotions without judgement, E‐PAtS supports healthier emotional regulation, which benefits both parents' well‐being and their child's emotional development (Aydin 2023; Zimmer‐Gembeck et al. 2022).

### 4.3 Using Emotional Journals as a Measure: Research and Clinical Considerations

Most studies on psychological well‐being focus primarily on its cognitive component and often neglect subjective well‐being and its emotional component (Frantz et al. 2018; National Institute for Health and Care Excellence 2016; Shepherd et al. 2020). A previous study on E‐PAtS highlighted the value of adding coping strategies to the program that could be practised in the present moment (Gore et al. 2022). The emotional journal could serve a dual purpose (i.e., as both a research and clinical tool). Asking parents to take a moment to focus on what is happening inside them can both enrich the evaluation of the program's outcomes and enhance participants' emotional and psychological well‐being.

Incorporating a subjective measure, such as an emotional journal, into program evaluation provides a more intimate view of participants' lived experiences and, as a mindfulness prompt, can also act as a small clinical intervention. Self‐reports of current emotional experiences tend to be more valid than those made retrospectively (Robinson and Clore 2002). However, these measures may be influenced by social desirability and require that individuals have the skills to accurately identify and articulate their emotions. Given the complexity of emotions, which encompasses cognitive, emotional, and behavioural dimensions, relying on a single measurement instrument can be challenging.

It is important to point out that only 10 parents completed all 16 entries in the emotional journal. Inasmuch as developing emotional regulation skills is not an explicit or direct goal of the E‐PAtS program, it is perhaps unsurprising that some parents opted out of the experience. Future iterations of the program could include dedicated components that gradually introduce and support emotional awareness and regulation skills that would make participants feel more comfortable engaging with these reflective exercises. Additionally, in terms of evaluating the use of emotional journals, supplementing it with qualitative data through open‐ended responses or additional interviews could provide richer insights into the emotional experiences of parents, and a mixed‐methods approach could be considered for future research to better contextualise and deepen the understanding of the quantitative changes reported.

### 4.4 Positive Intervention Approaches and Acceptance

Historically, research in mental health has focused predominantly on psychological dysfunction, with limited studies examining positive psychological functioning. Mental health is often equated with the absence of illness rather than the presence of wellness. Such perspectives overlook essential human capacities for flourishing and the protective benefits associated with overall well‐being (Ryff and Singer 1996). There is growing support for incorporating measures of both mental health symptom and well‐being, as these two inversely related but distinct constructs may call for separate intervention strategies (Bohlmeijer and Westerhof 2021; Jankowski et al. 2020).

An approach centered on validating emotional experiences prioritises enhancing overall well‐being rather than simply alleviating suffering (Seligman et al. 2004). This perspective was fundamental to the development of the E‐PAtS program and remains a crucial theme in discussions among parents during sessions (Gore et al. 2022). By acknowledging and exploring a wide range of emotional experiences, both comfortable and uncomfortable, E‐PAtS encourages parents to share their feelings and strategies for managing challenges. This validation fosters a supportive environment that helps parents navigate their emotions and ultimately promotes healthier emotional development in their children. In relation to the logic model and underlying mechanisms for E‐PAtS, it is important to note that an increase in both comfortable and uncomfortable emotional experiences would not necessarily be interpreted as a negative outcome. Rather, a rise in the identification of emotions, regardless of their valence, could indicate enhanced emotional awareness, a key component of emotional regulation and a step toward acceptance (Hayes et al. 1999; Kashdan et al. 2015). From this perspective, the present findings, which suggest a potential increase in positive emotional experiences, are encouraging. However, different results could have equally demonstrated that E‐PAtS supports parents' emotional and psychological well‐being by strengthening their emotional skills and fostering greater awareness and acceptance of their internal experiences.

The theoretical stance underlying the E‐PAtS logic model closely echoes the processes of acceptance as conceptualised in Acceptance and Commitment Therapy (ACT; Hayes et al. 1999), in that it provides a space for parents to observe their emotions and experiences without judgement. Although the development of the E‐PAtS logic model might not explicitly be based on ACT components, several elements within the program's approach and content encourage similar shifts in perspective for parents. Discussions within sessions try to emphasise what is most important for the parent and their family to foster greater adaptation and coping skills. Similar to the ACT approach, E‐PAtS prioritises embracing uncomfortable internal experiences, rather than attempting to eliminate them through avoidance or control strategies. It also emphasises recognising and building on the positive aspects of parents' reality, while enhancing their ability to respond more flexibly by fostering an open mindset (Gore et al. 2022). In this context of exchanges promoting openness and acceptance, the approach reflects the concept of psychological flexibility central to ACT (Hayes et al. 1999). Psychological flexibility, as in the ability to accept difficult emotions and thoughts while remaining aligned with one's values and goals (Hayes et al. 1999), is crucial, as low levels of flexibility are linked to higher symptoms of depression, anxiety, and stress (Sairanen et al. 2019). By providing parents with not only evidence‐based strategies to support their child but also a space to share their emotions and build resilience, initiatives like E‐PAtS might help them navigate their own challenges while better supporting their children's emotional and developmental needs.

### 4.5 Limitations

This study has several limitations. First, the emotional journal required participants to engage in introspection and emotion recognition, which may have been challenging for some. Providing more guidance or examples might have improved usability. Second, as a self‐report measure, the journal was susceptible to social desirability bias, and the use of a three‐point Likert scale may have limited the precision of intensity ratings. Third, few participants completed all 16 entries, suggesting that the tool may not have been feasible or engaging for all. This attrition limits the representativeness and generalisability of the data. Fourth, the smaller sample size did not support more stringent controls to limit type I errors in multiple comparisons; the statistical effects noted should therefore be regarded as preliminary observations pending replication in a larger‐scale trial. Lastly, as emotional regulation was not a core goal of E‐PAtS, the journal should be viewed as an exploratory addition. Future research should further assess its clinical value and gather participant feedback to refine its integration.

## 5 Conclusion

This study aimed to investigate the emotional journey of parents with children who have intellectual and developmental disabilities as they participated in a support group program designed to enhance their emotional and psychological well‐being. By the end of the program, parents reported more comfortable emotions and fewer uncomfortable emotions. The first two sessions, which focused on access to formal services and promoting personal self‐care, yielded the most pronounced differences in the number of emotions expressed and a marked decrease in uncomfortable emotions. By facilitating self‐reflection and conversations with supportive peers, E‐PAtS has demonstrated the potential to help parents to better navigate and express their lived emotional journeys. This study underscores the relevance of using subjective measures to document parents' internal experiences when evaluating interventions aimed at supporting emotional and psychological well‐being.

## Ethics Statement

This research project was reviewed and approved by the Ethical Committee of the participating public agencies. All procedures were carried out in accordance with their guidelines and regulations.

## Consent

All written consents were obtained from participants for their participation in the program as well as in the research project.

## Conflicts of Interest

The authors declare no conflicts of interest.

## Supporting information


**Data S1:** Supplementary Information.

## Data Availability

The datasets generated and analysed during the current study are not publicly available due to confidentiality agreements and the sensitive nature of the data but are available from the corresponding author upon reasonable request.

## Endnotes

^1^
The term ‘intellectual and developmental disability’ (IDD) refers to conditions identified in early childhood that involve significant delays in key area of development, such as cognitive, language, motor domains, as well as adaptive behaviours and socio‐emotional development. It includes children with an official diagnosis, those awaiting a diagnosis, and those exhibiting atypicalities requiring developmental support (see Tassé et al. 2025 for a discussion on terminology).
