# Supplementary material for: Parents' Emotional Journey Throughout Their Participation in a Well‐Being Support Group Intervention
Source: J Appl Res Intellect Disabil. 2025 Aug 7;38(4):e70107. doi: 10.1111/jar.70107 (PMC12329625; doi:10.1111/jar.70107)
Supplement: Supplementary file 1 — Data S1: Supplementary Information. [file JAR-38-e70107-s001.docx]

**Supplementary material**

**Emotional journal**

**HOW DO YOU FEEL RIGHT NOW?**

Take a moment to check in with yourself and observe how you feel. Please identify all the emotions you are currently experiencing using the list below. You can also write them in the "Other emotion" boxes.

Feel comfortable sharing your emotions without judgment, there are no right or wrong answers. Once your answers are compiled, they will be denominated, so it will be impossible to identify you.

- Tired
- Shameful
- Happy
- Confident
- Angry
- Alone
- Curious
- Interested
- Disappointed
- Powerless
- Soothed
- Enthusiastic
- Sad
- Discouraged
- Satisfied
- Grateful
- Worried
- Motivated
- Optimistic
- Embarrassed
- Other emotion 1: ______________
- Other emotion 2: ______________

Please rate the intensity of the emotions you are feeling using the scale provided:

Please **only** indicate the intensity of the **emotions previously ticked**.

|  | A little | Enough | A lot |
| --- | --- | --- | --- |
| Tired | ⃝ | ⃝ | ⃝ |
| Happy | ⃝ | ⃝ | ⃝ |
| Angry | ⃝ | ⃝ | ⃝ |
| Curious | ⃝ | ⃝ | ⃝ |
| Disappointed | ⃝ | ⃝ | ⃝ |
| Soothed | ⃝ | ⃝ | ⃝ |
| Sad | ⃝ | ⃝ | ⃝ |
| Satisfied | ⃝ | ⃝ | ⃝ |
| Worried | ⃝ | ⃝ | ⃝ |
| Optimistic | ⃝ | ⃝ | ⃝ |
| Shameful | ⃝ | ⃝ | ⃝ |
| Confident | ⃝ | ⃝ | ⃝ |
| Alone | ⃝ | ⃝ | ⃝ |
| Interested | ⃝ | ⃝ | ⃝ |
| Powerless | ⃝ | ⃝ | ⃝ |
| Enthusiastic | ⃝ | ⃝ | ⃝ |
| Discouraged | ⃝ | ⃝ | ⃝ |
| Grateful | ⃝ | ⃝ | ⃝ |
| Motivated | ⃝ | ⃝ | ⃝ |
| Embarrassed | ⃝ | ⃝ | ⃝ |
| Other emotion 1 | ⃝ | ⃝ | ⃝ |
| Other Emotion 2 | ⃝ | ⃝ | ⃝ |
